# 改良IA “3+4”方案与中剂量阿糖胞苷方案巩固治疗低危急性髓系白血病的疗效比较

**DOI:** 10.3760/cma.j.issn.0253-2727.2022.07.011

**Published:** 2022-07

**Authors:** 霄虹 刘, 晋松 贾, 婧 王, 立众 宫, 菲菲 唐, 文静 于, 晓璐 朱, 昱 王, 倩 江, 英军 常, 晓甦 赵, 国瑞 阮, 亚溱 秦, 晓军 黄, 浩 江

**Affiliations:** 北京大学人民医院、北京大学血液病研究所，国家血液系统疾病临床医学研究中心，北京 100044 Peking University People's Hospital, Peking University Institute of Hematology, National Clinical Research Center for Hematologic Disease, Beijing 100044, China

对于首次完全缓解（CR）、适合标准化疗的低危急性髓系白血病（AML）患者，中外指南推荐中、大剂量阿糖胞苷（Ara-C）2～4个疗程或自体造血干细胞移植作为巩固治疗[1]–[3]。有研究显示，细胞遗传学低危AML患者采用多个化疗药物联合的化疗方案和单一大剂量Ara-C比较，无病生存（DFS）及总生存（OS）差异无统计学意义[4]–[5]。2019年Ara-C全球供应不足，米托蒽醌断药，结合既往研究，我中心制定改良IA“3+4”方案替代中剂量Ara-C（MD-Ara-C）作为AML缓解后巩固治疗方案。现回顾性分析本中心37例于2019年接受改良IA“3+4”方案巩固治疗2～4个疗程低危AML患者，以81例2017–2018年接受MDAra-C方案患者为对照组，比较两组巩固治疗的疗效，报告如下。

## 病例与方法

1. 病例：纳入2017年1月至2019年12月在北京大学人民医院血液病研究所诊治的初治低危AML患者118例。所有患者均经骨髓细胞形态学、流式细胞术免疫分型、细胞遗传学和分子生物学检查，根据WHO2016造血与淋巴组织肿瘤分类标准[6]进行诊断。AML预后危险分层根据2017年欧洲白血病网（ELN）关于成人AML的推荐进行[1]。符合以下条件的患者纳入本研究：①ECOG评分≤2分，初治低危AML，基因分型为RUNX1-RUNX1T1阳性或NPM1^mut^/FLT-ITD^wt/low^或CBFβ-MYH11阳性；②诱导化疗1个疗程达CR者；③巩固治疗采用2～4个疗程MD-Ara-C或者改良IA “3+4”方案者。本研究为回顾性研究，改良IA“3+4”组为2019年病例，MD-Ara-C组为2017–2018年符合入组条件的历史对照病例。主要研究终点为巩固治疗2个及4个疗程时主要分子学反应（MMR），次要研究终点为16个月无复发生存（RFS）率及总生存（OS）率。入选患者均签署了知情同意书。

2. 治疗方案：诱导化疗方案包括采用“3+7”方案（IA、DA）、HAA或CAG方案。

缓解后给予2～4个疗程MD-Ara-C方案（Ara-C 2 g/m^2^，每12 h 1次，第1～3天），或者2～4个疗程改良IA“3+4”方案（去甲氧柔红霉素 6 mg/m^2^，第1～3天；Ara-C 300 mg/m^2^，第1～4天），之后持续化疗患者接受DA、AA、EA、CAG等方案化疗，巩固治疗共5～7个疗程。NPM1伴FLT3-ITD^low^突变、CBF-AML伴C-KIT突变患者，根据2017年NCCN指南AML危险分层高危、中危[3]，在接受至少2个疗程巩固治疗后行异基因造血干细胞移植（allo-HSCT）；巩固治疗2个疗程后RUNX1-RUNX1T1、CBFβ-MYH11融合基因和NPM1^mut^转录本水平较自身诊断时基线水平下降<3个对数级者，有allo-HSCT条件并根据患者意愿选择allo-HSCT[7]–[9]。具体移植方案详见本中心既往报道[10]。

3. 基因检测方法及微小残留病（MRD）评估时间：基因检测方法详见既往报道[11]–[13]，RUNX1-RUNX1T1、CBFβ-MYH11融合基因和NPM1^mut^以ABL基因作为内参，目标基因水平（％）＝（目标基因拷贝数/ABL基因拷贝数）×100％。MRD评估时间为诱导缓解治疗后的每疗程巩固治疗前，期间有病情变化随时评估骨髓。

4. 疗效评估标准及定义：根据2017年ELN关于成人AML的推荐进行CR、复发、RFS、OS的评估[1]。MMR：RUNX1-RUNX1T1、CBFβ-MYH11融合基因和NPM1^mut^转录本水平较自身诊断时基线水平下降>3个对数级。粒细胞缺乏持续时间：停化疗出现中性粒细胞<0.5×10^9^/L到恢复至>0.5×10^9^/L的持续时间。PLT<20×10^9^/L持续时间：停化疗后出现PLT<20×10^9^/L到恢复至>20×10^9^/L的持续时间。HGB<70 g/L持续时间：停化疗出现HGB<70 g/L到恢复至>70 g/L的持续时间。

5. 随访：通过住院病例查阅、门诊及电话随访，随访时间截至2020年12月1日。

6. 统计学处理：采用SPSS 22.0软件进行统计学分析。两组间率的比较采用卡方检验；连续变量采用两独立样本*t*检验及Mann-Whitney非参数检验［采用*M*（*P*_25_，*P*_75_）表示］；OS和RFS分析采用Kaplan-Meier法，并进行Log-rank检验；*P*值采用双侧分析，*P*<0.05为差异有统计学意义。累积复发率（CIR）采用竞争风险模型R软件分析。

## 结果

一、患者确诊时基本资料

纳入118例患者，其中改良IA“3+4”组37例（IDA：善唯达22例，艾诺宁15例），MDAra-C组81例。改良IA“3+4”组：男22例，女15例；中位年龄43（16～66）岁；RUNX1-RUNX1T1阳性者16例，合并C-KIT阳性者3例；CBFβ-MYH11阳性者4例，合并C-KIT阳性者1例；NPM1^mut^者17例，合并FLT3-ITD^low^者7例。MDAra-C组：男43例，女38例；中位年龄42（16～68）岁；RUNX1-RUNX1T1阳性者33例，合并C-KIT阳性者13例；CBFβ-MYH11阳性者15例，合并C-KIT阳性者3例；NPM1^mut^者33例，合并FLT3-ITD^low^者4例。两组基线情况差异无统计学意义（*P*值均>0.05）。

二、两组巩固治疗2个及4个疗程MMR比较

1. 两组巩固治疗2个疗程MMR比较：改良IA“3+4”组37例患者，巩固治疗2个疗程获得MMR者36例（97.3％），未获得MMR者1例（2.7％）。MD-Ara-C组81例患者，巩固治疗2个疗程获得MMR者68例（84％），未获得MMR者13例（16％），两组差异无统计学意义（*χ*^2^＝4.327，*P*＝0.062）。

2. 巩固治疗4个疗程MMR比较：改良IA“3+4”组完成4个疗程巩固治疗者20例，均获得MMR，中位随访时间15.75（12.7～22.5）个月。MDAra-C组完成4个疗程巩固治疗者36例，中位随访时间32.5（12～45）个月，其中1例NPM1^mut^/FLT3-ITD^low^患者在巩固2个疗程后获得MMR，完成4个疗程MDAra-C治疗后失去MMR，余35例均获得MMR。两组差异无统计学意义（*χ*^2^＝0.566，*P*＝1.000）。

三、allo-HSCT情况

118例患者，47例（39.8％）行allo-HSCT（17例为同胞全合allo-HSCT，30例为半相合allo-HSCT），中位年龄37（16～60）岁，男28例，女19例。改良IA“3+4”组10例（27.0％）行allo-HSCT，中位allo-HSCT时间为9（4～18.5）个月；MDAra-C组37例（45.6％）行allo-HSCT，中位allo-HSCT时间为8.5（4～25）个月。CR_2_期行allo-HSCT患者6例（改良IA“3+4”组1例，MD-Ara-C组5例）。CR_1_期行allo-HSCT患者41例，其中根据2017年中外AML指南，危险分层为中高危者23例（改良IA“3+4”组7例，MDAra-C组16例）；因巩固治疗2个疗程后未获得MMR行allo-HSCT者6例，均为MDAra-C组；余12例中，10例因后续治疗中失去MMR行allo-HSCT（改良IA“3+4”组2例，MD-Ara-C组8例），2例MDAra-C组患者因患者意愿行allo-HSCT。

四、复发及生存情况

1. 巩固治疗2个疗程后获得MMR患者复发及生存情况：改良IA“3+4”组获得MMR患者36例（97.3％，36/37），中位随访时间为15.75（7.7～24.5）个月；复发3例（8.3％），中位复发时间10.5（5.5～13.5）个月。MDAra-C组获得MMR患者68例（84.0％，68/81），中位随访时间为32.5（5～46.5）个月；复发15例（22.1％，15/68），中位复发时间12.0（3.5～21.0）个月，其中1例NPM1^mut^/FLT3-ITD^low^患者为allo-HSCT后复发。改良IA“3+4”组和MD-Ara-C组16个月RFS率分别为（87.3±6.1）％和（79.1±5.0）％（*P*＝0.337），OS率分别为（97.2±2.7）％ 和（94.0±2.9）％（*P*＝0.296），差异无统计学意义。

2. 全部患者复发及生存情况：改良IA“3+4”组37例患者，中位随访时间为16.0（7.7～24.5）个月；复发3例（8.1％），中位复发时间10.5（5.5～13.5）个月。MD-Ara-C组81例患者，中位随访时间为37.0（5.0～46.5）个月；复发18例（22.2％），中位复发时间11.5（3.5～21.0）个月。改良IA“3+4”组和MD-Ara-C组患者1年CIR分别为5.6％和12.4％（*P*＝0.250）。两组16个月RFS率分别为（87.7±5.9）％和（78.7±4.6）％（*P*＝0.262），OS率分别为（97.3±2.7）％和（92.4±3.0）％（*P*＝0.211），差异均无统计学意义。

改良IA“3+4”组只接受化疗患者27例，中位随访时间为14.7（4.0～21.7）个月。MD-Ara-C组只接受化疗患者44例，中位随访时间为16.0（4.0～45.0）个月。两组1年RFS率分别为（96.3±3.6）％ 和（81.8±5.8）％（*P*＝0.182），OS率分别为100％和（95.3±3.2）％，差异无统计学意义（*P*＝0.143）。

改良IA“3+4”组37例患者中死亡1例（allo-HSCT后发生移植相关死亡）。MD-Ara-C组81例患者中死亡18例（9例因血液学复发死亡，9例allo-HSCT后发生移植相关死亡）。两组均无化疗相关死亡。

五、血液学及非血液学不良事件

共92例患者可随访到巩固治疗1个疗程期间血液学及非血液学不良反应，其中改良IA“3+4”组28例，MD-Ara-C组64例。血液学方面，两组ANC<0.5×10^9^/L和HGB<70 g/L持续时间差异无统计学意义（*P*值均>0.05）。MD-Ara-C组PLT<20×10^9^/L持续时间明显长于改良IA“3+4”组，血小板输注量明显多于改良IA“3+4”组，差异有统计学意义（*P*值均<0.001）（表1）。

**表1:** 改良IA“3+4”和MD-Ara-C组患者巩固治疗1个疗程后血液学不良反应［*M*（*P*_25_，*P*_75_）］

组别	例数	ANC<0.5×10^9^/L持续时间（d）	HGB<70 g/L持续时间（d）	PLT<20×10^9^/L持续时间（d）	红细胞输注量（U）	血小板输注量（U）
改良IA“3+4”组	28	5（4，5）	0（0，1）	2（2，4）	0（0，2）	1（1，2）
MD-Ara-C组	64	4.5（4，5）	0（0，1）	4（3，5）	0（0，2）	2（1，2.75）

*z*值		−0.164	−1.194	−4.002	−1.498	−3.537
*P*值		0.870	0.232	<0.001	0.134	<0.001

注：IA：去甲氧柔红霉素+阿糖胞苷；MD-Ara-C：中剂量阿糖胞苷；ANC：中性粒细胞绝对计数

感染方面，患者出现感染性发热、肺部感染、泌尿系感染、肛周感染及血流感染等之一者，即视为发生感染。改良IA“3+4”和MD-Ara-C组感染发生率分别为39.3％和67.2％，差异有统计学意义（*χ*^2^＝6.255，*P*＝0.012）。

## 讨论

中外AML指南及共识均推荐中大剂量Ara-C 2～4个疗程作为初治低危AML缓解后的巩固治疗。细胞遗传学低危且获得CR的AML患者，中大剂量Ara-C联合蒽环或蒽醌类药物作为巩固方案耐受性好，RFS/DFS和OS明显延长[14]–[15]。我所既往研究显示，对于老年AML患者，MA是安全有效的巩固治疗方案[16]。2019年Ara-C全球供应不足，米托蒽醌断药，结合既往研究，本中心制定改良IA“3+4”方案替代MD-Ara-C作为AML缓解后巩固治疗方案。

多项研究证实，诱导化疗后及巩固治疗期间不同时间点的骨髓MRD水平可影响生存[17]–[18]。在RUNX1-RUNX1T1、CBFβ-MYH11及NPM1^mut^ AML中骨髓MRD水平对预后的判断意义已被证实[19]–[21]。我所前期研究显示，在RUNX1-RUNX1T1、CBFβ-MYH11及NPM1^mut^ AML中，巩固治疗2个疗程后骨髓MRD水平下降3个对数级与良好生存相关[7]–[9]。本研究显示，改良IA“3+4”和MD-Ara-C巩固2个疗程后骨髓MRD水平下降>3个对数级者分别为97.3％和84％，差异无统计学意义。表明改良IA“3+4”和MD-Ara-C作为初治低危AML首次缓解后治疗可获得同样的近期疗效。

Koistinen等[15]的研究结果指出，中大剂量Ara-C联合蒽环或蒽醌类药物作为巩固治疗可延长细胞遗传学低危AML患者RFS（4年RFS率为70％）和OS（5年OS率在低危、中危/正常核型、中危/异常核型及高危组分别为71％、47％、37％、8％），减少Ara-C剂量并不影响RFS和OS。本研究发现，初治低危AML获得CR后采用改良IA“3+4”和MD-Ara-C巩固治疗2～4个疗程者比较，16个月RFS率和OS率差异无统计学意义，两组1年的CIR差异亦无统计学意义。提示改良IA“3+4”方案和MD-Ara-C巩固治疗在低危AML中有同样的远期疗效。

巩固治疗期间增加去甲氧柔红霉素的累积剂量可改善无白血病生存，并且不增加感染及非血液学不良反应[22]。本研究结果也显示，初治低危AML获得CR后采用改良IA “3+4”方案巩固治疗未增加血液学不良反应，改良IA“3+4”组辐照机采血小板输注量及感染发生率均较MD-Ara-C组降低。

综上所述，初治成人低危AML（RUNX1-RUNX1T1阳性、CBFβ-MYH11阳性及NPM1^mut^/FLT3-ITD^wt/low^）首次缓解后巩固治疗方案的选择可根据药物供应及患者意愿等情况选择改良IA“3+4”方案或者MD-Ara-C，两组近期疗效相当，血液学不良反应相似，且改良IA“3+4”方案可减少血小板输注，降低感染发生率，为低危AML缓解后治疗提供一个新选择。本研究为回顾性研究，样本量小，观察时间不够长，有局限性，确切的结论需通过前瞻性研究进一步证实。

## References

[1] Döhner H, Estey E, Grimwade D (2017). Diagnosis and management of AML in adults: 2017 ELN recommendations from an international expert panel[J]. Blood.

[2] 中华医学会血液学分会白血病淋巴瘤学组 (2017). 成人急性髓系白血病(非急性早幼粒细胞白血病)中国诊疗指南(2017年版)[J]. 中华血液学杂志.

[3] O'Donnell MR, Tallman MS, Abboud CN (2017). Acute Myeloid Leukemia, Version 3.2017, NCCN Clinical Practice Guidelines in Oncology[J]. J Natl Compr Canc Netw.

[4] Miyawaki S, Ohtake S, Fujisawa S (2011). A randomized comparison of 4 courses of standard-dose multiagent chemotherapy versus 3 courses of high-dose cytarabine alone in postremission therapy for acute myeloid leukemia in adults: the JALSG AML201 Study[J]. Blood.

[5] Moore JO, George SL, Dodge RK (2005). Sequential multiagent chemotherapy is not superior to high-dose cytarabine alone as postremission intensification therapy for acute myeloid leukemia in adults under 60 years of age: Cancer and Leukemia Group B Study 9222[J]. Blood.

[6] Arber DA, Orazi A, Hasserjian R (2016). The 2016 revision to the World Health Organization classification of myeloid neoplasms and acute leukemia[J]. Blood.

[7] Qin YZ, Xu LP, Chen H (2015). Allogeneic stem cell transplant may improve the outcome of adult patients with inv(16) acute myeloid leukemia in first complete remission with poor molecular responses to chemotherapy[J]. Leuk Lymphoma.

[8] Zhu HH, Zhang XH, Qin YZ (2013). MRD-directed risk stratification treatment may improve outcomes of t(8;21) AML in the first complete remission: results from the AML05 multicenter trial[J]. Blood.

[9] 赵 婷, 主 鸿鹄, 王 婧 (2017). 早期评估NPM1突变阳性急性髓系白血病患者残留白血病水平的预后意义[J]. 中华血液学杂志.

[10] Wang Y, Wu DP, Liu QF (2014). In adults with t(8;21)AML, posttransplant RUNX1/RUNX1T1-based MRD monitoring, rather than c-KIT mutations, allows further risk stratification[J]. Blood.

[11] Ruan GR, Li JL, Qin YZ (2009). Nucleophosmin mutations in Chinese adults with acute myelogenous leukemia[J]. Ann Hematol.

[12] Qin YZ, Zhu HH, Jiang Q (2014). Prevalence and prognostic significance of c-KIT mutations in core binding factor acute myeloid leukemia: a comprehensive large-scale study from a single Chinese center[J]. Leuk Res.

[13] 秦 亚溱, 李 金兰, 主 鸿鹄 (2007). 实时定量RT-PCR技术测定初治白血病患者常见融合基因转录子水平及其标准化的探讨[J]. 中华血液学杂志.

[14] Liu J, Mi Y, Fu M (2009). Intensive induction chemotherapy with regimen containing intermediate dose cytarabine in the treatment of de novo acute myeloid leukemia[J]. Am J Hematol.

[15] Koistinen P, Räty R, Itälä M (2007). Long-term outcome of intensive chemotherapy for adults with de novo acute myeloid leukaemia (AML): the nationwide AML-92 study by the Finnish Leukaemia Group[J]. Eur J Haematol.

[16] 王 婧, 江 滨, 江 浩 (2016). 老年急性髓系白血病小剂量MA与CAG方案诱导治疗疗效比较及预后影响因素分析[J]. 中华血液学杂志.

[17] Schuurhuis GJ, Heuser M, Freeman S (2018). Minimal/measurable residual disease in AML: a consensus document from the European LeukemiaNet MRD Working Party[J]. Blood.

[18] Boddu P, Gurguis C, Sanford D (2018). Response kinetics and factors predicting survival in core-binding factor leukemia[J]. Leukemia.

[19] Ivey A, Hills RK, Simpson MA (2016). Assessment of Minimal Residual Disease in Standard-Risk AML[J]. N Engl J Med.

[20] Jourdan E, Boissel N, Chevret S (2013). Prospective evaluation of gene mutations and minimal residual disease in patients with core binding factor acute myeloid leukemia[J]. Blood.

[21] Yin JA, O'Brien MA, Hills RK (2012). Minimal residual disease monitoring by quantitative RT-PCR in core binding factor AML allows risk stratification and predicts relapse: results of the United Kingdom MRC AML-15 trial[J]. Blood.

[22] Bradstock KF, Link E, Di Iulio J (2017). Idarubicin Dose Escalation During Consolidation Therapy for Adult Acute Myeloid Leukemia[J]. J Clin Oncol.

